# Supplementary material for: Nash Up, Virus Down: How the Waiting List Is Changing for Liver Transplantation: A Single Center Experience from Italy
Source: Medicina (Kaunas). 2022 Feb 14;58(2):290. doi: 10.3390/medicina58020290 (PMC8874675; doi:10.3390/medicina58020290)

SUPPLEMENTARY MATERIALS

Figure S1. Annual trend of waitlist registrations between 2006 and 2019. The calendar year 2020 was not considered, since the study collected data until 06.2020

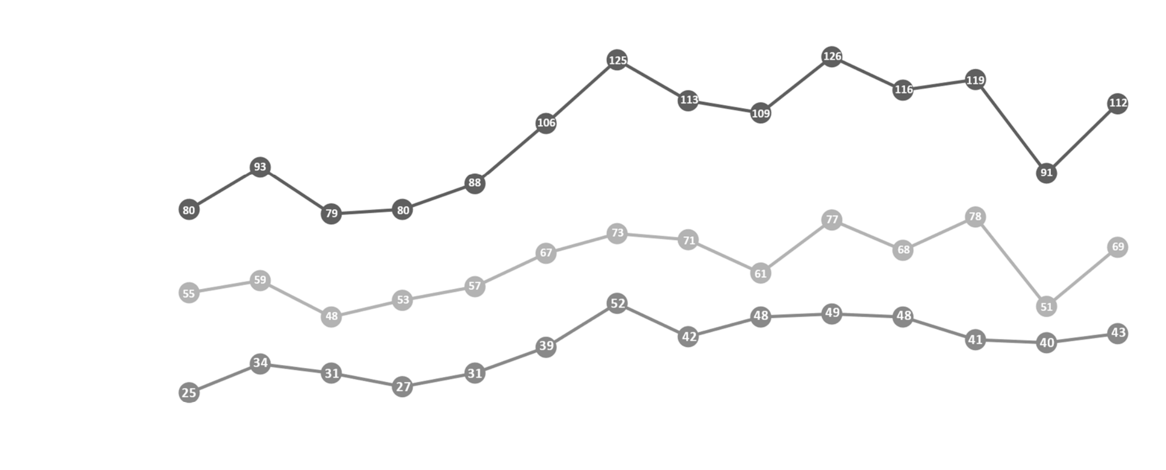

	2006	2007	2008	2009	2010	2011	2012	2013	2014	2015	2016	2017	2018	2019
overall	80	93	79	80	88	106	125	113	109	126	116	119	91	112
Decompensated	55	59	48	53	57	67	73	71	61	77	68	78	51	69
HCC	25	34	31	27	31	39	52	42	48	49	48	41	40	43

Figure S2. Post-transplant survival between NASH patients according to the primary indication (dec-NASH vs. HCC-NASH).

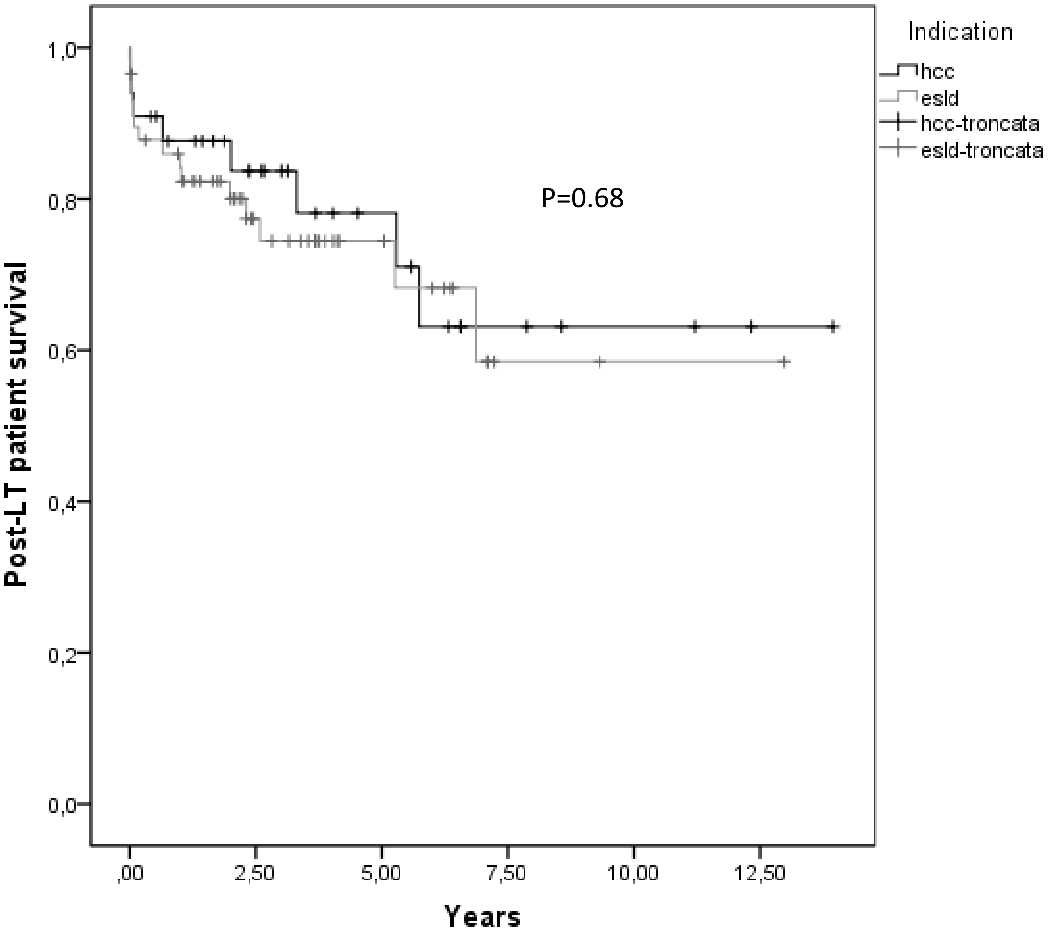

Supplement: Supplementary file 1 [file medicina-58-00290-s001.zip › medicina-1511140-supplementary.pdf]
